# Proceedings Terrell Medical Society

**Published:** 1890-11

**Authors:** 


### Society Notes.

### PROCEEDINGS TERRELL MEDICAL SOCIETY.

Dr. James Orr, President; Dr. E. E, Smiley, Secretary and Treasurer. Regular meetings, first and third Mondays of each month.

Dr. F. S. White said, owing to the fact that irregular practitioners of medicine were attempting to join local medical societies which were auxiliary to the State Medical Association, steps must be taken whereby our constitution should be so amended, as to coincide with that of the State Medical Association and exclude any and all who are not regular graduates of medicine; that societies which admit such and are members of the State Medical Association, are liable to be excluded from affiliating as members of that body. As Dr. White favored purity in the ranks of medicine, and the attainment of higher medical proficiency, he saw fit to offer a resolution to the effect that the Constitution of the Terrell Medical Society be so amended as will make the requirements for membership same as the State Medical Association.

Drs. Smiley, Strain, Wallace and a large number of our members endorsed the views as set forth by Dr. White.

Dr. Orr took a different view of the matter and held that such a course would do injustice to the undergraduates who were members of this society; that to cast them out, would have a tendency to array them against the regular physician, and so discourage them that instead of attempting to advance and reach that degree of skill and proficiency so much to be desired, would lapse into a state of injured indifference, and it would result in greater disaster to the medical profession than to permit them becoming members of local societies; said that he espoused the cause of the man who was striving to obtain wisdom; that he was once an undergraduate and that he had been made to feel the cuts from regular physicians; that there was nothing to be gained from associating with the State Mediccal Association; all the benefit derived from it, that he could see, was the privilege of wearing a little piece of blue ribbon and paying our $5 annual dues, and nothing more; that our society had prospered and that we could continue so to prosper without any connection with the State Medical Association.

Dr. Wallace said that it was impossible for our society to retain an organic relation with the State Medical Association and permit undergraduates and irregular practitioners to be enrolled as its members; that it would be in direct violation of the rules of eligibility to membership, as defined by the State Medical Association; that the articles of the constitntions of societies admitted as auxiliaries must conform to the same articles as are found embodied in the constitution of the State Medical Association; that in discussing the merits of the Texas State Medical Association he was bound to say, it equalled, if not surpassed in grandeur, dignity and greatness any State medical association or college of medicine in the Union; that any one and especially a physician, who had failed to see and appreciate the benefits to be derived from such an august assembly of intelligent physicians, other than wearing a piece of blue ribbon and paying his annual dues, certainly had his commiseration.

Dr. Fry then unsheathed his sword and hastened to the front, extolling the benefits derived and to be derived from association by auxiliary societies from such an embodiment of wisdom and medical lore as is represented in the State Medical Association; favored raising our standard,—that in so doing the truly meritorious, the ardent and zealous student would press on all the more vigorously to be worthy of having his name enrolled among the distinguished physicians of the State of Texas.

The action required a two-thirds vote to amend or alter the constitution, which being put before the society, prevailed, and as a consequence the names of those who were members and not regular graduates, were dropped from its list of members. Hence it will be observed that the Terrell Medical Society favors a high and dignified plane of professional ability.

